# Glaucoma Diagnosis with Machine Learning Based on Optical Coherence Tomography and Color Fundus Images

**DOI:** 10.1155/2019/4061313

**Published:** 2019-02-18

**Authors:** Guangzhou An, Kazuko Omodaka, Kazuki Hashimoto, Satoru Tsuda, Yukihiro Shiga, Naoko Takada, Tsutomu Kikawa, Hideo Yokota, Masahiro Akiba, Toru Nakazawa

**Affiliations:** ^1^R&D Division, Topcon Corporation, Tokyo, Japan; ^2^Cloud-Based Eye Disease Diagnosis Joint Research Team, RIKEN Center for Advanced Photonics, RIKEN, Wako, Japan; ^3^Tohoku University Graduate School of Medicine, Sendai, Japan; ^4^Image Processing Research Team, RIKEN Center for Advanced Photonics, RIKEN, Wako, Japan

## Abstract

This study aimed to develop a machine learning-based algorithm for glaucoma diagnosis in patients with open-angle glaucoma, based on three-dimensional optical coherence tomography (OCT) data and color fundus images. In this study, 208 glaucomatous and 149 healthy eyes were enrolled, and color fundus images and volumetric OCT data from the optic disc and macular area of these eyes were captured with a spectral-domain OCT (3D OCT-2000, Topcon). Thickness and deviation maps were created with a segmentation algorithm. Transfer learning of convolutional neural network (CNN) was used with the following types of input images: (1) fundus image of optic disc in grayscale format, (2) disc retinal nerve fiber layer (RNFL) thickness map, (3) macular ganglion cell complex (GCC) thickness map, (4) disc RNFL deviation map, and (5) macular GCC deviation map. Data augmentation and dropout were performed to train the CNN. For combining the results from each CNN model, a random forest (RF) was trained to classify the disc fundus images of healthy and glaucomatous eyes using feature vector representation of each input image, removing the second fully connected layer. The area under receiver operating characteristic curve (AUC) of a 10-fold cross validation (CV) was used to evaluate the models. The 10-fold CV AUCs of the CNNs were 0.940 for color fundus images, 0.942 for RNFL thickness maps, 0.944 for macular GCC thickness maps, 0.949 for disc RNFL deviation maps, and 0.952 for macular GCC deviation maps. The RF combining the five separate CNN models improved the 10-fold CV AUC to 0.963. Therefore, the machine learning system described here can accurately differentiate between healthy and glaucomatous subjects based on their extracted images from OCT data and color fundus images. This system should help to improve the diagnostic accuracy in glaucoma.

## 1. Introduction

Glaucoma is a chronic, neurodegenerative ocular disease characterized by optic neuropathy and visual disturbance that corresponds to optic disc cupping and optic nerve fiber degeneration [1]. Lowering the intraocular pressure (IOP) is an effective, evidence-based treatment for open-angle glaucoma (OAG) [2, 3]. This treatment requires early diagnosis and adequate IOP control to maintain a good quality of life. This becomes even more important in today's aging societies.

Generally, glaucomatous structural changes precede functional changes. Therefore, the early diagnosis of glaucoma relies on detecting these structural changes. The most basic diagnostic tool for glaucoma diagnosis is the analysis of color fundus images, which can identify glaucomatous optic neuropathy, including rim thinning and notching, undermining, cupping, a high cup-to-disc ratio, disc hemorrhage, and retinal nerve fiber layer (RNFL) defects. Another powerful tool is optical coherence tomography (OCT), which can be used to describe glaucoma both qualitatively and quantitatively [4]. OCT, which targets the optic disc and macular area, can reveal preperimetric glaucoma with high sensitivity and specificity [5]. For glaucoma diagnosis, the power of different OCT scan parameters, such as disc topography, circumpapillary RNFL thickness (RNFLT), macular RNFLT, ganglion cell layer plus inner plexiform layer thickness, and ganglion cell complex (GCC) layer thickness, differs with variations in glaucomatous structural changes [6]. Therefore, for diagnosing all types of glaucoma, it is best to use OCT data both from the disc and the macula.

Recently, machine learning technologies and deep learning, in particular, have seen dramatic progress and has enabled the development of new algorithms to automate eye disease diagnosis [7, 8], including glaucoma screening based on color fundus images [9, 10] and OCT data [11, 12]. However, the proposed machine learning models in these studies dealt only with either kind of images to distinguish glaucoma patients from healthy subjects, which is quite different from the actual clinical diagnosis by ophthalmologists. In other words, there have been only a few machine learning models reported using multimodality images relevant to glaucoma diagnosis.

In this study, we aimed to build a machine learning classification model that combines the information of color fundus and OCT data from the macula and disc area to objectively classify glaucomatous and healthy eyes and to evaluate its performance of detecting early glaucoma.

## 2. Materials and Methods

### 2.1. Subjects and Extracted Images

This study enrolled 208 eyes of 208 OAG patients, including 49 preperimetric glaucoma subjects and 149 eyes from 149 healthy subjects, to use exactly one eye of each participant. The study adhered to the tenets of the Declaration of Helsinki, and the protocols were approved by the institutional review board of RIKEN (Wako3 26-4).

All eyes were diagnosed by three ophthalmologists, who were unaware of each other's diagnosis, and each eye was labeled either as glaucoma or healthy by unanimous decision. OAG was diagnosed according to the presence of the following: (1) glaucomatous optic neuropathy with corresponding visual field defects, (2) abnormally reduced circumpapillary retinal nerve fiber layer thickness (cpRNFLT), and (3) an open angle in a gonioscopic examination. The exclusion criteria were as follows: (1) best-corrected visual acuity less than 20/25, (2) high myopia (i.e., an axial length longer than 27.0 mm), and (3) the presence of ocular diseases other than OAG or of systemic diseases affecting the visual field. Mean deviation (MD) values were obtained with the Humphrey visual field analyzer using the Swedish interactive threshold algorithm standard strategy of the 24-2 program. Only reliably measured data were used (i.e., with a fixation loss <20%, false-positive errors <15%, and false-negative errors <33%). A glaucomatous visual field was defined, according to the Anderson-Patella criteria [13], by one or more of the following: (1) a cluster of three points with probabilities of <5% on the pattern deviation map in at least one hemifield (including ≥ 1 point with probability of <1% or a cluster of two points with a probability of <1%), (2) glaucomatous hemifield test results outside the normal limits, or (3) a pattern standard deviation (SD) beyond 95% of normal limits, as confirmed in at least two reliable examinations. Control subjects presented OCT measurements in both disc and macula within the normal range, no history of ocular or systemic diseases affecting the visual field, no elevated IOP, and no apparent sign of a glaucomatous optic disc.

The sex distribution as well as the mean values with SDs for age, IOP, MD, and axial length is shown in Table 1. There is no significant difference between healthy and OAG patients in the sex ratio (*p*=0.081 for chi-square test) or age (*p*=0.214 for *t*-test), but the axial length is significantly increased in the OAG group (*p* < 0.0001 for *t*-test).

**Figure 1:**
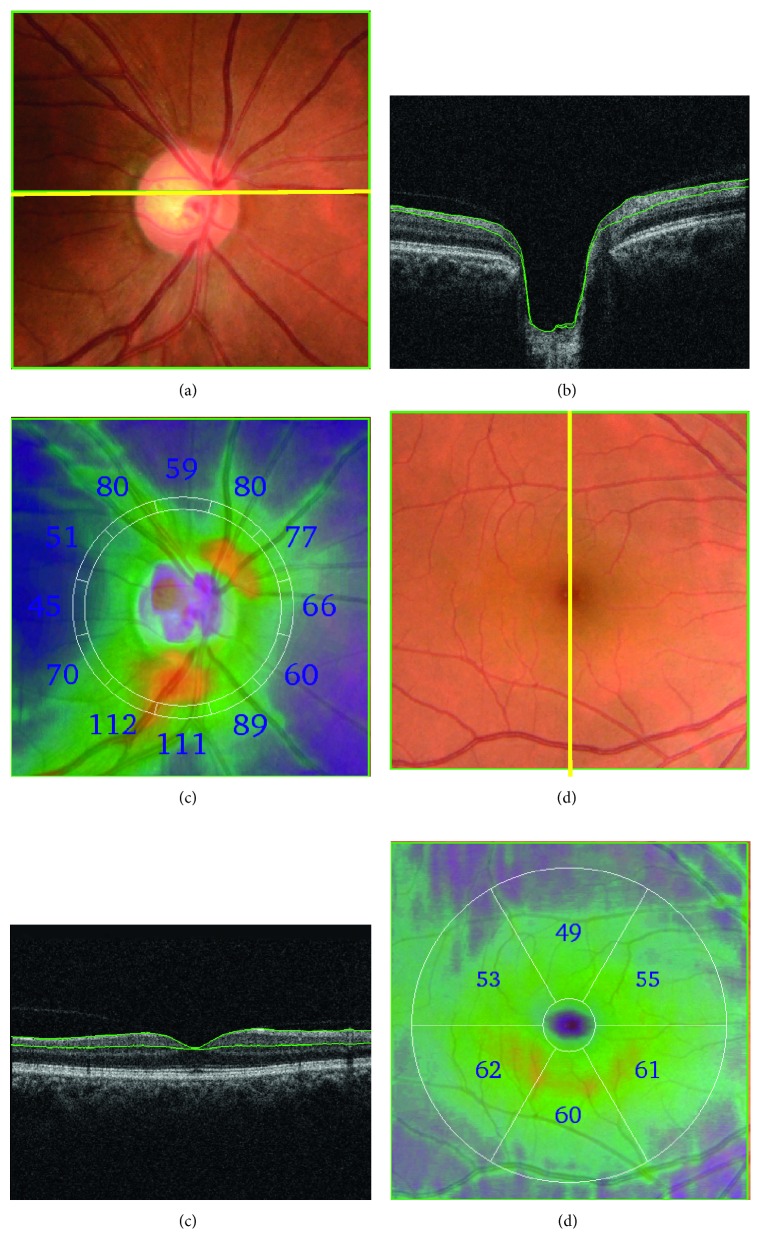
Optical coherence tomography (OCT) images. (a) A color fundus photo of the optic disc area. (b) Cross-sectional OCT image at the yellow line in (a) where green lines in (b) show the detected layer information for calculating the retinal nerve fiber layer (RNFL) thickness. (c) RNFL thickness map, where the numbers indicate the thickness in micrometers in 12 sectors around the optic disc. (d) A color fundus photo of the macular area. (e) Cross-sectional OCT image at the yellow line in (d) where green lines in (e) show the detected layer information calculating the ganglion cell complex (GCC) layer thickness. (f) GCC thickness map, where the numbers indicate the thickness in micrometers in 6 sectors around the fovea at the center of the macular area.

All participants were additionally examined with spectral-domain OCT (3D OCT-2000, Topcon Corp., Tokyo, Japan), using a vertical macular scan (7 mm × 7 mm, 512 A-scans × 128 frames) and a horizontal disc scan (6 mm × 6 mm, 512 A-scans × 128 frames). Both types of OCT scan were analyzed with Topcon's OCT analysis software (FastMap Ver. 8.40) to identify the boundaries of the RNFL and GCC (Figure 1). Besides the layer boundaries, the fovea and the disc center were also automatically detected with the same software and verified by the ophthalmologists.

Macular GCC thickness maps were created from these OCT scans using an RGB color 224 × 224-pixel format. To create deviation maps, the macular GCC thickness maps were down-sampled to a 30 × 30 grid in which thickness values were subtracted from the average of the overall dataset from the healthy subjects used in Topcon's reference database and then translated into a grayscale image. Similarly, from the disc OCT data, disc RNFLT maps were created in an RGB color 224 × 224 -pixel format. Then, a 5.2 mm × 5.2 mm area centered on the disc center was down-sampled to a 26 × 26 grid. To create deviation maps, the mean value of the healthy subjects in Topcon's reference database was calculated, subtracted from the down-sampled RNFLT map and translated into a grayscale image. Additionally, RGB color fundus images captured with disc fixation were cropped manually to their central 7 mm × 7 mm area with a resolution of 768 × 768 pixels, and their green channel was extracted and saved after normalization as a grayscale image in a 224 × 224-pixel format. Color fundus images of the macular area were not used in this study because it is difficult to acquire important information from a fundus photo of the macular area since there are only a few points of interpretation to diagnose glaucoma clinically.

**Figure 2:**
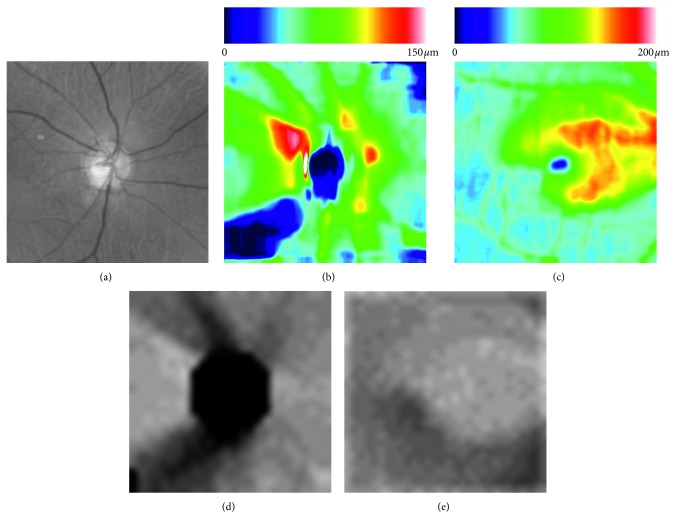
Example of extracted images for machine learning. (a) Fundus image centered at the optic disc in grayscale format. (b) Disc RNFL thickness map. (c) Macular GCC thickness map. (d) Disc RNFL deviation map. (e) Macular GCC deviation map.

Five different kinds of images were employed by our machine learning system. They are shown in Figure 2(a), the fundus image centered at the disc in grayscale format; Figure 2(b), the disc RNFL thickness map; Figure 2(c), the macular GCC thickness map; Figure 2(d), the disc RNFL deviation map; and Figure 2(e), the macular GCC deviation map.

### 2.2. Transfer Learning of the Convolutional Neural Network

A convolutional neural network (CNN) is a supervised classifier based on deep learning [14]. It is comprised of several convolutional layers as well as subsampling layers, good at designing powerful filters to retrieve the sensitive image features for the classification task. In this study, we adopted the CNN architecture VGG19 [15], a CNN model with 19 layers being widely used to solve image classification problems. To classify healthy and glaucomatous eyes, the output layer of VGG19 was changed into a new softmax layer with two units suitable for this task. On the other hand, transfer learning is a machine learning method to apply a developed model for previous tasks to a new task domain. Based on this strategy, we fine-tuned a VGG19 pretrained on the ImageNet large-scale visual recognition challenge dataset as the starting point, to build a classification model for each kind of images with stochastic gradient descent (SGD) optimization. In total, five different VGG19 models were built. Dropout and data augmentation including horizontal flip, random rotation, and random shift were used during training. The VGG19 models were transfer learned based on an SGD optimization method with momentum 0.9 and a fixed learning rate of 10^−5^ for 100 epochs. In this study, a system with Ubuntu 16.04 OS and a single GTX 1080 TI was used, consuming about 80 minutes to train models for a kind of input images.

### 2.3. Proposed Approach

**Figure 3:**
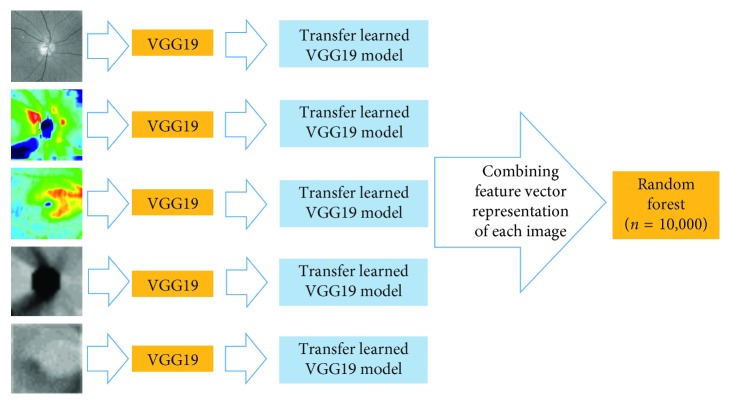
Proposed approach.

Final clinical judgments of a disease are always performed by ophthalmologists after they interpret various types of clinical data and images. Similarly, to incorporate the results from each model, a random forest (RF; tree number = 10,000) was trained to classify the images of healthy and glaucomatous eyes, combining the 4,096-dimensional feature vector representation of each input image, resulting from removing the second fully connected layer. Thus, five subordinate CNN classification models were trained separately: (1) a VGG19 model based on disc fundus images centered at optic disc, (2) a VGG19 model based on disc RNFL thickness maps, (3) a VGG19 model based on macular GCC thickness maps, (4) a VGG19 model based on disc RNFL deviation maps, and (5) a VGG19 model based on macular GCC deviation maps (Figure 3).

The evaluation of the classification performance relied on the area under curve of receiver operating characteristic (AUC) of the 10-fold cross validation (CV). Furthermore, a fixed random seed was used when splitting data for the cross validation, to allow reemploying the same images as training and test data.

## 3. Results

In this report, we describe a new machine learning algorithm for diagnosing glaucoma based on OCT-derived data, including disc fundus images as well as thickness and deviation maps of the macula and the optic disc. The 10-fold cross-validated AUCs for diagnosing glaucoma were 0.940 for disc fundus images, 0.942 for disc RNFL thickness maps, 0.944 for macular GCC thickness maps, 0.949 for disc deviation maps, and 0.952 for macular deviation maps. Additionally, we found that the10-fold CV AUC of this system rose to 0.963 when all classification models were combined (Table 2), while the 10-fold CV AUCs were 0.953 for images from disc OCT data, 0.954 for images from macular OCT data, 0.959 for images from the disc combined with the fundus image, 0.961 for the combination of disc fundus images, disc RNFL thickness maps, and macular GCC thickness maps, and 0.963 for images from OCT data.

## 4. Discussion

In this study, the machine learning system had an excellent ability to differentiate healthy and glaucomatous eyes, with a 10-fold CV AUC of 0.963. This system should, therefore, help to improve standards for glaucoma diagnosis and improve the diagnosis consistency. We found that transfer learning of VGG19 models is a suitable machine learning method to automate the differentiation between healthy subjects and glaucoma patients based on OCT-derived data. Supervised learning approaches have been most frequently used to discriminate between glaucomatous and nonglaucomatous eyes, and most published studies in the field of glaucoma research employ supervised machine learning techniques to improve diagnoses [16]. Belghith et al. calculated in their study the AUC to differentiate glaucoma with 0.91 for a Bayesian network, 0.69 for a neural network, and 0.6 for a support vector machine [17]. With a deep feed-forward neural network, the visual field of patients with preperimetric glaucoma can be distinguished from the visual field of healthy subjects with very high accuracy (AUC: 0.926) [18]. Furthermore, there have been reports that the accuracy reaches 0.98 when age, IOP, central corneal thickness, cpRNFLT, and the visual field are all considered [19]. Thus, the accuracy of diagnosis was higher with various kinds of measuring data. In the current study, it is notable we achieved an AUC value of 0.963 using only objective OCT data and disc fundus images without considering visual field data.

In this study, additional experiments were performed to validate the current system. For comparison, we also extracted ocular parameters, in total 140, as shown in Table 3. A commercial software commonly used in clinics quantified these parameters, to build an RF model (tree number = 10,000). Among these parameters, the average thickness was calculated in each grid, with fixed size and shape based on the automatically detected disc and macula center.

**Figure 4:**
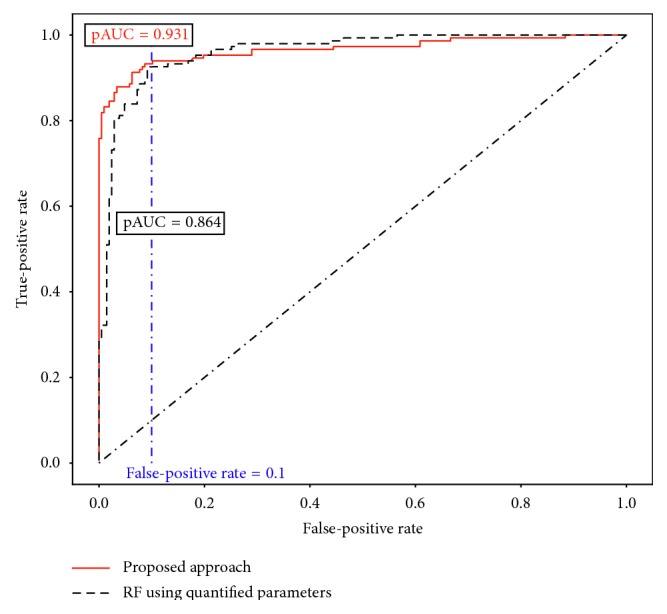
Comparison of glaucoma detection models with partial AUC.

As a result, the 10-fold CV AUC was 0.958 ± 0.030. Furthermore, a Wilcoxon signed rank sum test was used to statistically compare the models. We found that there is no substantial difference (*p*=0.053) between this RF model using additionally extracted ocular OCT parameters and our proposed RF using five different types of images. However, it is more important to consider the false-positive rate than the false-negative rate for an automatic model to detect a disease; thus, we applied a partial AUC (pAUC) [20] to evaluate our proposed model. When using the maximum of false-positives as a fixed rate of 0.1, our proposed model achieved a pAUC of 0.931 ± 0.055, which is significantly higher (*p*=0.048) than the pAUC of the RF using additional quantified parameters (pAUC: 0.864 ± 0.010; Figure 4). If the sensitivity was set to zero by adjusting the threshold, in other words, detecting all glaucoma cases including the 49 preperimetric glaucoma cases (MD: 0.14 ± 1.13), the specificity of our model was 0.789, while that of the RF using additional quantified parameters was 0.289.

**Figure 5:**
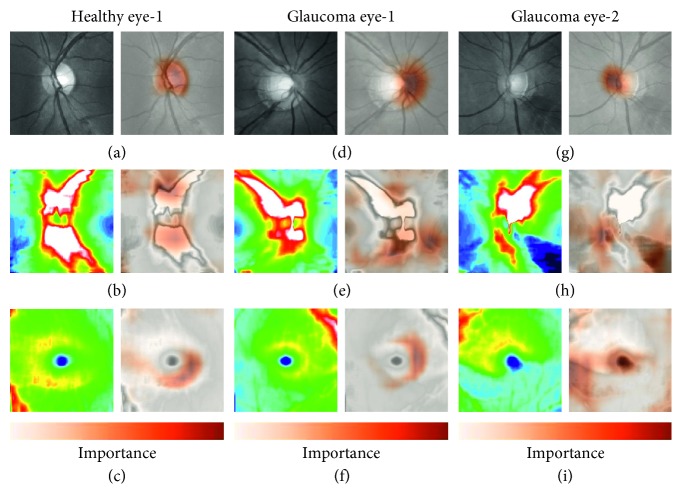
Visualization of the important areas in our VGG19 model. One healthy eye and two representative glaucoma eyes were randomly selected to show the area of interest in the input images. Results from a healthy subject (a, b, c) and glaucoma subject 1 (d, e, f) and 2 (g, h, i), showing class-discriminative regions in grayscale disc fundus images, disc RNFL thickness maps, and macula GCC thickness maps, respectively. Dark orange regions correspond to high scores for the diagnosis.

Furthermore, there is no statistically significant difference (*p*=0.061) between this RF model and RF using both thickness maps and disc fundus images, without using deviation maps. In other words, our proposed approach achieved the same classification performance, without automatically detecting the disc and macula center with image processing methods. Although the quantified parameters by the commercial software were evaluated and have been published online as a whitepaper (http://www.topcon.co.jp/eyecare/handout), the detection of disc centers fails in some myopic eyes, especially those with tilted optic discs, resulting in an inaccurate glaucoma quantification [21]. Since our proposed CNNs do not use the disc center detection, their classification performance would be unaffected by a failure to detect the centers of disc and fovea. Thus, our proposed machine learning model might be more robust to distinguish glaucoma patients from healthy subjects.  Figure 5 shows example images, demonstrating that the neural network was successfully able to identify pathologic regions on OCT images with grad-CAM [22]. These areas represent the area in each image most critical to the trained network in categorizing the image as glaucoma. The highlighted color maps show a tendency to favor thinner regions in the disc area and thicker regions in the macula. Measurements of the disc RNFL show that the area surrounding the optic disc is the thickest region; clinically, doctors usually examine RNFL thickness in the defect area. Furthermore, in macular GCC thickness maps, the peripheral area of the GCC is normally thinner than the central area. Moreover, the shape of the thick GCC area changes from a circular to a distorted structure as glaucoma progresses, making this area a good differentiator between healthy subjects and those with early glaucoma [23]. Thus, the CNN utilized areas that are characteristically used by doctors for glaucoma diagnosis.

We found that thickness and deviation maps of the macula and RNFL differed in their ability to discriminate between glaucoma patients and control subjects. The vulnerable zone in the macula and the lower temporal area of the disc in the cpRNFL is most susceptible to damage in glaucoma. Previously, we confirmed that approximately 75% of normal tension glaucoma patients have early-stage glaucoma damage in the macular vulnerability zone and the 6, 7, and 11 o'clock sectors of the cpRNFL [24, 25]. Although recent research has shown that damage in the macular vulnerability zone is a more sensitive indicator of early glaucoma than damage in the cpRNFL [6, 23], this is also reflected in the present study with better performance when using images from the macular area.

Limitations of this study include a relatively small study population, which may have affected the statistical power of our analyses. However, the model was evaluated with cross validation AUC, and our classification system should, therefore, be accurate to detect glaucoma subjects with an MD range of (−11.7, 3) dB. Second, the study used a cross-sectional design and included only subjects of a single ethnicity. The color of fundus photographs and the value of the OCT-measured RNFLT differ between ethnic types, although there is less variation in OCT data than in disc fundus images. Furthermore, our previous research [26] showed that the shape of the optic disc varies in different countries. Optic disc shape influences the susceptible area of the cpRNFL [26], the progression speed [27], and the relationship between structural and functional measurements [28]. Therefore, there is a need for additional, international validation of our machine learning method for different ethnic backgrounds in future studies. A third limitation arose from the significantly different spherical equivalent values in the study groups. Specifically, the glaucoma group included more cases with myopia, with an average axial length of 25.57 ± 1.53 mm. It is difficult to classify myopia and glaucoma subjects since myopia is a major risk factor for the presence of glaucoma in Asian subjects [29]. The appearance of the optic disc is significantly different in these patients, and the fundus photography shows usually a tilted disc with crescent peripapillary atrophy. On the other hand, the OCT data (both thickness and deviation data) were not significantly influenced by the presence of mild myopia in the subjects, and the CNN still recognized the areas characteristic of glaucomatous change in the disc and macular OCT thickness maps. Future studies enrolling larger numbers of myopic patients with and without glaucoma are needed in order to confirm the effectiveness of this machine learning algorithm.

## 5. Conclusions

In conclusion, this study used fundus images and extracted quantified images from OCT data, either alone or in combination, as the basis for an automated, objective, machine learning method for glaucoma diagnosis. Our combination method achieved an AUC of 0.963; it has the potential to be more sensitive to detect glaucoma in its early stages. Therefore, our findings should help to make more accurate glaucoma diagnoses, leading to a better daily clinical glaucoma care.

## Figures and Tables

**Table 1:** Demographic data.

	Healthy (*n*=149)	Glaucoma (*n*=208)	*p* Value
Sex (male/female)	80/69	179/108	>0.05
Age (years)	49.8 ± 15.9	51.6 ± 11.9	>0.05
Mean deviation (dB)	−0.21 ± 1.15	−3.90 ± 3.80	<0.0001
Axial length (mm)	23.97 ± 0.93	25.57 ± 1.53	<0.0001

**Table 2:** The AUC of the machine learning models alone and in combination.

Number	Cases	AUC (mean ± SD)
#1	Disc fundus image (green channel)	0.940 ± 0.039
#2	Disc RNFL thickness map	0.942 ± 0.037
#3	Macular GCC thickness map	0.944 ± 0.032
#4	Disc deviation map	0.949 ± 0.030
#5	Macular deviation map	0.952 ± 0.029
#6	Combination of #2 and #4 (images from disc OCT data)	0.953 ± 0.032
#7	Combination of #3 and #5 (images from macular OCT data)	0.954 ± 0.031
#8	Combination of #1, #2, and #4 (images from disc OCT data with fundus image)	0.959 ± 0.031
#9	Combination of #1, #2, and #3 (automatically detected disc and macular center were not used in creating images)	0.961 ± 0.029
#10	Combination of #2, #3, #4, and #5 (images from OCT data)	0.963 ± 0.030
#11	Combination of all images	0.963 ± 0.029

**Table 3:** Ocular parameters extracted from OCTs.

Number	Quantification type	Features
1	cpRNFLT average thickness from disc area OCT	Average cpRNFLT
2–5		cpRNFLT (quadrants)
6–11		cpRNFLT (6 sectors)
12–23		cpRNFLT (clockwise sectors)

24	Optic disc shape parameters from disc area OCT	Disc area
25		Cup area
26		Rim area
27		Cup volume
28		Rim volume
29		Cup/disc ratio (area)
30		Horizontal cup/disc ratio
31		Vertical cup/disc ratio
32		Horizontal disc diameter
33		Vertical disc diameter

34	GCC average thickness from macula area OCT	Average GCC thickness
35–40		GCC thickness (6 sectors)
41–140		GCC thickness (10 ∗ 10 grids)

## Data Availability

The images and diagnosis data used to support the findings of this study have not been made available because they are real clinical data from subjects visiting the hospital, and the subjects' right to privacy should be protected since it is possible to identify people from these data.
